# Frequent visitors at the psychiatric emergency room – A literature review

**DOI:** 10.1007/s11126-017-9509-8

**Published:** 2017-03-29

**Authors:** Manuela Schmidt

**Affiliations:** 1 Department of Nursing, Kristianstad University, 291 88 Kristianstad, Sweden; 2 Department of Health Sciences, Lund University, 221 00 Lund, Sweden

**Keywords:** Frequent visitor, Psychiatric emergency, Review, SWOT

## Abstract

Frequent visitors at the psychiatric emergency room (PER) constitute a small subgroup of patients, yet they are responsible for a disproportionate number of visits and thus claim considerable resources. Their needs are often left unmet and their repetitive visits reflect their dissatisfaction as well as that of PERs' staff. Motivated by these dilemmas, this study systematically reviews the literature about frequent visitors at PER and seeks to answer two questions: What characterizes frequent visitors at PER in the literature? and What characterizes PER in the literature? Based on 29 studies, this paper offers answers to the two questions based on a strength weakness opportunities and threats (SWOT) analysis. The results of the review and subsequent analysis of the literature revealed the multiplicity and complexity of frequent visitors' characteristics and how they appear to converge. Commonalities were more difficult to identify in PER characteristics. In some cases, this happened because the characteristics were poorly described or were context specific. As a result, it was not easy to compare the studies on PER. Based on SWOT and the findings of the analysis, the paper proposes new venues of research and suggests how the field of mental health might develop by taking into account its opportunities and threats.

## Introduction

Psychiatric emergency rooms (PER) have been changing as rapidly as the entire mental health care system. A strong impact on the change and development of PER had the initiation of the deinstitutionalization [1, 2] that started in the 1960s on a global scale. This soon resulted in fewer inpatient beds and outpatient services, more admissions, shorter stays, service gaps, as well as inadequate housing and community support services for those suffering from mental health problems; thus contributing to a growing number of mental health patients and a greater need for PER. In addition, an increase in substance abuse in general [3, 4], as well as the increasing occurrence of community social problems and decreasing tolerance for them [1] were additional reasons of the rapid evolution of PER. As an ultimate consequence of growing need and simultaneous cutbacks, PER have become an important gate keeper for providing care by use of triage [5]. PER are characterized by ongoing, chronic crisis management [2] while at the same time also attempting to provide specialized, high quality frontline care for patients when resources that are already scarce have to cover an increasing number of new and returning patients. For many who lack access to other sources of care or resources, PER have turned into safety nets [6, 7].

The social and economic burden of mental ill health is vast and continues to grow. Thus, in regard to PER, it becomes apparent that the growing discrepancy between patients’ needs and emergency resources [8, 9] contributes to both patients’ and staff members’ dissatisfaction which also could be a result of the lack of consensus concerning PER’s goals and resource allocation [9].

One could assume that persons visiting PER are a homogenous group or in Bachrachs words “*faceless representations of a homogeneous group”* [10] consisting of mainly those struggling with problems related to substance abuse or those who have been diagnosed with severe mental illnesses such as schizophrenia. However, visitors to PER are a very heterogeneous group of individuals with a wide spectrum of mental health issues [e.g. 11, 12, 13]: problems in living, social problems, and problems related to societal matters that are influenced by current events such as a terror attacks, wars or an influx of refugees. One distinct subgroup of patients in PER are frequent visitors [e.g. 3]. Even though they are a relatively small group, they account for a disproportionate high number of visits and thus claim a considerable amount of the already limited resources [5, 11, 13–16]. Frequent visitors at PER are fragile and exposed and because of the psychiatric nature of their illness they have difficulties expressing and demanding their rights in terms of mastering life in general and tackling psychiatric care in particular. Due to their repetitive behavior, it can be concluded that their needs are complex and varying and are insufficiently met by PERs. In 1983, Bassuk had already concluded that overutilization of PER mirrors the gaps in the health care system [17]. PER are the context in which acute crisis situations are taken care of, which means that staff is pressed to make fast decisions of short-term relevance. While this orientation might be satisfactory for persons that visit a PER once or twice, the expectations and needs of frequent visitors might be different. The fact that they return to PER indicates that they might need to be provided with a long-term solution and continuity of care [18, 19] that would decrease or end their visits to PER. Further, it indicates that a long-term sustainable development of PER themselves is required. The fact that frequent visitors are often referred to as difficult patients [2, 20] or as hard to treat [18] is another indication why they need to be given more attention. Since PER are part of the *human sector* where people work with and for other people [21], humane condition should be the core element in PER so as to reduce patients suffering and provide sustainable working settings for staff.

## Frequent visitors

The number of visits to PER are soaring not only due to deinstitutionalization but also due to increases in substance abuse [3] and in mental health problems in general. Previous studies have shown that PER are increasingly used by visitors with non-urgent needs [22–24], implying that the quality and availability of care for visitors suffering an acute crisis is being compromised [24]. These findings are in line with other studies that have pointed out that a number of visits of persons to PER have increased among those who do not represent “true” psychiatric emergencies but who use PER as a source of support [22, 23, 25]. Consequently, those non-urgent and frequent visits claim a disproportionate amount of PER’s resources [24] and put a substantial financial strain on PER [5]. Although PER represent a medical specialty, it can be concluded that they function as a “revolving door” for particularly persons who frequently visit PER [11, 26, 27] and who often have non-urgent mental health problems and needs [22].

- **Research question 1:** What characterizes frequent visitors at PER in the literature?

## Psychiatric emergency rooms

The field of health care and mental health care in particular have undergone significant changes over the past decades. As a consequence the same can be said about the development of PER and staff working in a psychiatric care setting that had to be adjusted in order to meet the new challenges.

PER is both time and staff-intensive [25]. Since the workload in PER is increasing [27] and utilization rates are soaring [28] it becomes more difficult for staff at PER to provide quality services. Central to the concept of psychiatric emergency is the subjective quality, the unpredictable nature of the emergencies, the wide range of diagnoses and symptoms of people visiting PER, lack of prior assessment or adequate planning, result uncertainty, severity, urgency etc. which puts many demands on staff at PER and makes PER a challenging workplace [29]. One consequence are inaccurate diagnoses that have shown to be associated with poor treatment outcomes and overuse of the most expensive types of services e.g. hospitalization [30].

Since the domain of PER is so far-reaching in terms of services that they provide and the variety of patients they serve, it is difficult to make a comprehensive evaluation of PER [1]. Since PER are the context where frequent visitors are comprehensively evaluated, their needs are assessed, and they are cared for, the characteristics and specific dimensions of PER are important to explore.

- **Research question 2:** What characterizes PER in the literature?

The aim of this paper is to explore what characterizes frequent visitors to PER and PER themselves by critically evaluating previous studies and providing a systematic review by addressing the two main research questions. Given the changing nature of PER’s frequent visitors and PER, this study provides an insight into the development of both over time. For each question, and inspired by Jackson et al. [31], a *SWOT* 1 analysis will be performed to consider the strengths and weaknesses of the previous research as well as opportunities and threats in it. The latter two aspects will lay the ground for suggestions for future research.

## Method

### Literature review

The papers relevant for this review were identified by a computer-based search done in the Web of Science and PubMed databases. A key word search was conducted by combining the search term “psychiatric emergency” with terms associated with a repetitive behavior or high frequency (“frequent”, “return”, “multiple”, “repeat”,” recurrent”,” high” or” increase”). The key word search was limited to the titles of articles. No time restrictions were imposed in order to cover as broad a field of research as possible.

### Inclusion criteria and selection process

The citations retrieved were scrutinized by reading the titles/abstracts/articles. The following inclusion criteria were used to identify studies to be included in the review; they had to be (1) published in a peer-reviewed journal in English; (2) based on original empirical data analysis; and (3) focused on frequent visitors in psychiatric emergency care settings.

## Results

In total, 67 items were identified in the Web of Science database and 23 in the by PubMed database. After duplicates were excluded, the total number of studies was 65. An additional 36 were excluded because they did not meet the inclusion criteria. Table 1 lists the author(s), year, data's origin, method, sample size and data of the remaining 29 articles.

**Table 1:** Summary of articles included

No	Authors (year)	Data’s origin	Method	Sample	Data
1	Raphling, D. L., & Lion, J. (1970) [36]	USA	mixed	15	Observations on FV + register data
2	Steer, R. A., Diamond, H., Litwok, E., & Henry, M. (1979) [37]	USA	quant	442	Home visits
3	Munves, P. I., Trimboli, F., & North, A. J. (1983) [38]	USA	quant	3824	Patients with no previous record at PER
4	Pérez, E., Minoletti, A., Blouin, J., & Blouin, A. (1986) [39]	Canada	quant	913 913	Questionnaires on patient filled in by staff Questionnaires filled in by patient
5	Surles, R. C., & McGurrin, M. C. (1987) [40]	USA	mixed	NM 27,899 35,250	Interviews with staff; Register data Register data (from other services)
6	Ellison, J. M., Blum, N. R., & Barsky, A. J. (1989) [41]	USA	quant	68 62	FV; Register data NFV; Register data
7	Hansen, T. E., & Elliott, K. D. (1993) [42]	USA		1144	Register data
8	Sullivan, P. F., Bulik, C. M., Forman, S. D., & Mezzich, J. E. (1993) [43]	USA	quant	16,257	Register data
9	Klinkenberg, W. D., & Calsyn, R. J. (1997) [44]	USA	quant	319	Register data
10	Spooren, D. J., Van Heeringen, K., & Jannes, C. (1997) [45]	Belgium	quant	13,323	Register data
11	Saarento, O., Hakko, H., & Joukamaa, M. (1998) [15]	Finland	quant	537	Patients with no previous record at PER
12	Saarento, O., Kastrup, M., & Hansson, L. (1998) [46]	Denmark/Finland	quant	1055	Patients with no previous record at PER
13	Segal, S. P., Akutsu, P. D., & Watson, M. A. (1998) [47]	USA	quant	417 NM	Register data; Brief questionnaire to staff
14	Arfken, C. L., Zeman, L. L., Yeager, L., Mischel, E., & Amirsadri, A. (2002) [3]	USA	quant	48 5722	Questionnaires with staff; Register data
15	Segal, S. P., Akutsu, P. D., & Watson, M. A. (2002) [26]	USA	quant	See 13	
16	Arfken, C. L., Zeman, L. L., Yeager, L., White, A., Mischel, E., & Amirsadri, A. (2004) [34]	USA	mixed	74 74 68	Interviews with FV; Register data Interviews with NFV; Register data Telephone interviews with family members
17	Bruffaerts, R., Sabbe, M., & Demyttenaere, K. (2005) [48]	Belgium	quant	531	Questionnaires filled in by staff about FV
18	Pasic, J., Russo, J., & Roy-Byrne, P. (2005) [16]	USA	quant	17,481	Register data
19	Ledoux, Y., & Minner, P. (2006) [11]	Belgium	quant	2470	Register data
20	Chaput, Y. J., & Lebel, M. J. (2007) [13]	Canada	quant	14,825	Register data
21	Chaput, Y. J., & Lebel, M. J. (2007) [5]	Canada	quant	19,740	Register data
22	Goldstein, A. B., Frosch, E., Davarya, S., & Leaf, P. J. (2007) [33]	USA	quant	509	Register data
23	Paradis, M., Woogh, C., Marcotte, D., & Chaput, Y. (2009) [49]	Canada	quant	4	PER
24	Boyer, L., Dassa, D., Belzeaux, R., Henry, J. M., Samuelian, J. C., Baumstarck-Barrau, K., & Lancon, C. (2011) [50]	France	quant	8860	Register data
25	Buus, N. (2011) [2]	Denmark	qual	11	Interviews with nursing staff
26	Aagaard, J., Aagaard, A., & Buus, N. (2014) [27]	Denmark	mixed	15 8034	15 interviews with FV; Register data
27	Richard-Lepouriel, H., Weber, K., Baertschi, M., DiGiorgio, S., Sarasin, F., & Canuto, A. (2015) [51]	Switzer-land	quant	4322	Register data
28	Lincoln, A. K., Wallace, L., Kaminski, M. S., Lindeman, K., Aulier, L., & Delman, J. (2016) [35]	USA	mixed	16 NM	Interviews with FV; Quantitative data
29	Nossel, I. R., Lee, R. J., Isaacs, A., Herman, D. B., Marcus, S. M., & Essock, S. M. (2016) [52]	USA	quant	97	Register data

*FV* frequent visitor

*NFV* non-frequent visitor

### Description of findings

Of the articles included in the review, 13 were published before 2000 (1970 to 2000) and 16 after 2000 (2001 to 2016). The articles were mainly published in journals within the fields of health care sciences, public, environmental and occupational health and psychiatry with very few exceptions in nursing, social work or emergency medicine. The majority of articles (14) was published in *Psychiatric Services*, which before 1995 was called *Hospital and Community Psychiatry*. All the journals are ranked and have impact factors varying between 0.769 and 5.605, as reported in Web of Science for 2015. Data for 16 of the articles was collected in USA, 9 in Europe and 4 in Canada, showing the clear dominance of the American research community in this field.

Twenty of the studies included had a quantitative approach focusing on register data of patients. Three additional quantitative studies concentrated on psychiatric emergency services, one on staff members, and another one on an implementation of an intervention. Out of 29 studies, one had a qualitative approach, interviewing staff about their categorization process of frequent visitors [2].

Five studies used a mixed method approach, combining interviews (or observations) of patients/nurses with register data. Out of 29 studies, one focused on a patient group below 18 years [33], whereas the remainder of 28 focused on the adult population, though this was not explicitly stated in many of the articles. None of the studies included explicit organizational/economic perspectives.

The quantitative data was analyzed using statistical tests, mainly regression analysis. In the qualitative study, discourse analysis was used with an underlying social constructionist framework. Furthermore, one article was a case control study [34], and another was a community based participatory research study [35].

### **Research question 1:** What characterizes frequent visitors at PER in the literature?

Table 2 is a summary of the definitions used in the literature reviewed, either as determined in the introduction or method section or as part of the results if some categorization was made there.

**Table 2:** Definition of frequent visitor from literature review

No	Examples of terms used	Conceptual definition of FV	Operationalization of the FV (examples of predictors of FV or FV are more likely to (be))
1	Patients with repeated admissions	NM	Attitude of helpless persons victimized by powerful external forces; place solution to their problems entirely in hands of physician; request for hospitalization and medication; demanding, provocative, and manipulative in communication with staff
2	Multiple visit; subsequent visits	NM	Displaying bizarre behavior during 1st visit; 92.2% receive same diagnosis as during 1st visit
3	Repeat patient visits	Return visit within 12 months after initial visit	Unemployed; need public assistance; have psychiatric history and/or, cognitive difficulties; severe primary diagnosis at initial visit
4	Non-repeaters Repeat users	- > 0 visits preceding index visit - > Group 1: 1 repeat visit within 6 months preceding index visit; Group 2: 2 or more repeat visit within 6 months preceding index visit	Group 1: Self-referred; single; have previous and current psychiatric treatment; schizophrenic; personality disorder; younger Group 2: Younger; unaccompanied; separated; comply more often with ambulatory follow-up; have poor rapport with staff in PER (no sig. Differences in sociodem and socioecon characteristics between NR and group 1 and 2)
5	Heavy users	Three or more admissions per year	Resemble young adult chronic patients; male, 17–35 years; schizophrenic; refuse outpatient care
6	Frequent repeaters	6 or more visits	Resident of catchment area; homicidal; self-injured; intoxicated during visits; absence of psychotherapist; have anxiety; self-referred; schizophrenic; have diagnosis of alcohol and/or substance abuse; borderline personality; concurrent psychiatric treatment; referred to outpatient treatment (no sign. Differences in sociodem. And socioecon. Characteristics)
7	Frequent repeaters Occasional repeaters Non-repeaters	- > 4–12 visits in a year - > 2–3 visits - > single visit	Schizophrenic; less often referred to outpatient clinics; visits after 4:30 p.m. and on weekends
8	Repeat users	4 groups: 1 visit, 2–4 visits, 5–10 visits and 11–162 visits	Male; younger; schizophrenic; suffer from major depression;, non-white; unmarried, unemployed; co-morbidity with substance abuse as 1st 2nd or 3rd diagnosis
9	Repeat visit; return visits	Repeat visit after 18 months	Previous psychiatric hospitalization; currently receiving outpatient treatment and not receiving aftercare
10	Repeated psychiatric referrals	NM	Male; younger; previous hospitalization; substance abuse disorder; inpatient treatment at end of visit
11	Repeat users	3–24 visits during the last 2 years of the follow-up	Male; living alone;, suffering from more serious diagnosis
12	Repeat users	Patients belonging to the upper tenth percentile of the emergency outpatient contacts, which in this study indicates at least three emergency contacts during the l-year follow-up	Location 1: male; divorced or unmarried; living with their parents; without their own housing; unemployed; age 25–44 Location 2: no sig.; differences in sociodem. Characteristics Have index contact with psychiatric services in outpatient care; self-referrals
13	Involuntary returnees and non-returnees	Return within 12 months	Spend more days in hospital after evaluation; psychotic disorder; more dangerous; less treatable; more insured; comply more with their referrals for treatment (no sign. Differences in sociodem. Characteristics),
14	Frequent visitors Infrequent visitors	- > 6 and more visits per calendar year - > 5 or less visits per calendar year	Motives for visits from staffs’ view: temporal pattern, weather, lunar variable
15	Involuntary returnees and non-returnees	Return within 12 months	See 13
16	Frequent visitors	6 or more visits in 12 months following index visit	Mon-adherent with treatment; admitted to inpatient hospital; homeless; rather drink than do drugs before visit; visit also other PER; have previous psychiatric hospitalization (no sign diff. in diagnosis) In interviews: report PER’s convenient location, no need for appointment, shelter, medication
17	Patients with recurrent utilization, repeated referrals	Patients with a history of PER visits	Female, mean age 37.3; lives with family; unemployed; have substance use disorders, personality disorders; noncompliant with aftercare; previous outpatient treatment
18	High utilizers	Three definitions: 1-patients with visits at least two standard deviations above the mean number of visits (selected because standard deviation units are the most common measure of variability); 2-patients with 6 or more visits in a single year (selection on the basis of previous studies); and 3- patients with 4 or more visits in one quarter (selected on the basis of the definition by the county).	Male; unemployed; enrolled in public mental health system; developmental disability; homeless; living dependently; have previous psychiatric hospitalization; schizophrenic
19	Frequent repeaters Occasional repeaters	- > 4 contacts or more during index period of 16 months - > 2 to 3 contacts during index period of 16 months	Male; younger; mean age 37.5; socially disabled; have psychosis; suffer from grief; self-referrals; lower welfare status
20	Patients who make multiple visits, Multiple visit patients	4 groups: 1 visit, 2 visits, 3–10 visits, 11+ visits	Younger; schizophrenic; have co-morbid psychiatric diagnosis; less dual diagnosis with substance abuse; more frequently placed under observation or hospitalized; unemployed
21	Multiple visit patients	intermediate group: 4–10 visits; heavy user group: 11+ visits	4–10 visits: schizophrenic; substance abuse; use of multiple services 11+ visits: schizophrenic; bipolar disorders; shorter time intervals between visits; use of multiple services
22	Repeat visitors	Repeat visit within six months after index visit	African American, show disruptive behavior; previous psychiatric hospitalization; suffer from diagnostic co-morbidity
23	Increase in utilization; utilization rates	NM	PER
24	Frequent visitors Occasional visitors	- > six or more visits in 6 years - > 2–5 visits in 6 years	Younger; single; homeless;, have non-affective psychotic disorders, schizophrenic, diagnostic variability
25	Frequent visitors	By nurses as persons who did not profit from psychiatric treatment and who could not mobilize sufficient resources in their psychosocial network to find alternatives to ER visits	Successful visits: relatively rapid with core problems and needs clearly identified; experience of reciprocity and rapport Difficult visits: nurses are not able to deal swiftly with visitor or/and visitor did not profit from treatment, discrepancies between assistance offered at PER and visitors’ own perception of their needs; not all FV were classified as difficult
26	Frequent visitor	5 visits or more per year	Schizophrenic; male; living in sheltering housing In interviews: final resort for help in an unbearable situation; integrated and valued part of social network
27	Recurrent visits (frequent visitors)	3 visits or more per year	Personality disorder (no sociodemographic predictors were found)
28	Repeat use of PES, frequent users, high utilizers, non-frequent users	Repeat use within 90 days	In interviews: instability in domains like housing, employment, finances, interpersonal relationships, formal treatment services, use of medication, living with substance abuse etc.
29	Frequent users	3 visits or more in a year	Implementation of a model to decrease use of PER and increase use of outpatient services

*FV* Frequent visitors

*NM* not mentioned

*NR* Non-Repeater

### Strengths

One strength of the literature is that most of the articles are based on large samples, which allows for generalization to the population. Several papers went beyond descriptive analysis and applied more sophisticated statistical techniques, such as logistic regression or linear regression analysis or both [e.g. 3, 5, 16, 27]. This leads to a more thorough understanding of the causal inference. Most of the studies used a longitudinal design, which showed how frequent visitors as a group change over time and allowed for better understanding of behavioral (proxied) patterns of that patient group. A few studies used new methodological approaches, e.g., mixed method designs [27, 34–36, 40]. This provides a better understanding for the complexity of frequent visitors as a group, no less from the perspective of significant others and nurses.

The descriptions of frequent visitors in the literature are another strength. On an operational level, frequent visitors were often described as being single [e.g. 39, 50], unmarried [e.g. 39, 43], homeless [e.g. 16, 34, 50], or living alone [e.g. 15, 16]. They were also found to have unreliable social support [e.g. 16] and be socially disabled [e.g. 11]. All these characteristics may be indicators of social isolation. Further, socioeconomic characteristics such as unemployment [e.g. 16, 39, 43] and economic impairment [e.g. 11, 48] have been found in the literature. Studies also have shown that the group mainly consists of men [e.g. 11, 15, 43] and persons of a young age [e.g. 11, 13, 43, 50]. Persons who frequently visit PER often suffer from a mental illness such as personality disorder [e.g. 16, 39, 46, 48] or schizophrenia [e.g. 5, 13, 39, 41] and substance abuse [e.g. 11, 41, 48]. They also are known for prior psychiatric hospitalization [e.g. 15, 16, 26, 33, 34, 39, 41, 44]. This substantial variation of characteristics indicates that this group has a rather heterogenic need profile, implying a complexity in supplying care for those persons. In summary, many variables are repetitive (e.g., diagnosis variables, which are found in almost all articles), whereas other variables are context-specific characteristics, like race or socioeconomic factors.

### Weaknesses

One weakness of the literature is the lack of current data. Though six studies were published after 2010, the data used in them was relatively dated; the latest study was published in 2016 using data from February 2009 to April 2010 [52]. This has left a gap in the years covered and a need for studies using data more collected recently so as to understand current developments. In addition, majority of the studies used a quantitative approach based on register data, suggesting a stronger focus is needed on more qualitative or mixed method designs that provide an opportunity to gain an in-depth understanding about frequent visitors. Basing studies on register data also meant that they lacked the perspectives on non-patients, such as organizational and staff perspectives or from significant others. Especially the perspective of the frequent visitors of PER might be important to consider, yet it is rarely taken into account when quantitative methods are used. Majority of the studies did not report practical implications and thus lacked relevance for praxis. The studies used many different definitions and terms for frequent visitors, which unnecessarily complicates comparison of the studies.

Though the diagnoses of frequent visitors was mentioned in most articles, they were described and analyzed in different ways (e.g., three diagnoses are given per visit or the most common diagnosis is picked or articles mention the principle diagnosis or severe primary diagnosis). This creates diagnostic confusion particularly in the context of longitudinal studies, which most of the studies are, and thus they lack transparency.

Additionally, only in few quantitative studies a control group was used [e.g. 5, 16, 34], which could be seen as a methodological weakness.

### Opportunities

One way of moving forward in the research area would be to develop the concept of frequent visitors further by conducting qualitative studies from which qualitative definitions of frequent visitors could be derived. This could allow for an in-depth understanding of frequent visitors and their needs, which would prepare the way for the development of effective interventions.

Another way forward would be to explore the different dimensions of the definition of frequent visitors, by exploring the views of different external actors. This might contribute to a more holistic definition because different perspectives would be taken into account.

Finally, one could study frequent visitors’ specific needs, life styles, behavior, and social networks in order to develop the interventions that are necessary and that could be implemented with a long-term perspective.

### Threats

There is a danger of misinterpreting the results of the studies if the settings, definitions, and terms are so varied. Because the majority of studies were conducted in US, the findings lack a broader perspective. Further, most of the studies missed taking the findings to a higher theoretical level. They did not explain their results by using the underlying theoretical reasons, but instead were driven mainly by empirical aims. Thus, although the studies succeeded in identifying patterns and characteristics of frequent visitors, they did not provide reasons for the results. As a consequence, the situation of frequent visitors would not be improved based on these studies because knowing only about their patients’ patterns is not enough. More focus should be paid to practical implications in order to make a difference in frequent visitors’ situations.

### **Research question 2:** What characterizes PER in the literature?

Categories “organizational context,” “geographical context,” “types of services and facilities, “processes and procedures,” “service usage,” “health care system,” and “staff” are shown below in Table 3. The characteristics of the PER were derived from the literature included in the review that were mainly described in the introduction and method sections of the articles.

**Table 3:** Characteristics of PER from literature review

No	Organizational context	Geographic context	Services and facilities	Processes and procedures	Service usage	Health Care System	Staff
1	General public hospital	Massachusetts	Walk-in clinic, admitted on unselected basis	Brief but thorough evaluation of psychiatric, social, and general medical conditions, follow up after discharge by other psychiatric facilities within hospital or by community agencies	NM	NM	NM
2	Home visiting team belong to general public hospital	NM	Mobile component of a 24 h a day	9-passenger station wagon with mobile telephone and pager system; description of primary and secondary goal; if protective environment needed court or voluntary commitment in accordance with law	Description of process, phone call, weapon involvement, police, reflection over violence and injury, description of intervention	NM	A psychiatric corpsman (trained by the navy) and a registered psychiatric nurse
3	University hospital	Dallas	24 h a day	Psychiatric interview record	NM	NM	Psychiatrist or psychiatric resident, a registered nurse, a clerk, and clinical psychology interns and medical students at specified times, under supervision
4	General hospital	Ottawa, catchment area 700.000	40 beds for psychiatry	Clinical assessment follows the guidelines of the initial evaluation form	NM	NM	NM
5	5 general hospitals, 1 private psychiatric hospital, 1 not affiliated with a hospital	7 PER in Philadelphia, in 5 different areas	24 h a day, provide 85% of PE care in the city	NM	NM	NM	NM
6	General hospital	Massachusetts	NM	NM	NM	NM	NM
7	NM	Portland	NM	NM	NM	NM	Nurse or resident daytime consultation Monday–Friday; otherwise, on-call resident
8	University hospital	Pittsburgh; socioeconomic status of pop varies	24 h a day; specialized sections	NM	NM	NM	NM
9	General public hospital	Metropolitan region of 2,000,000 inhabitants	24 h a day		Number of visits and admissions per year	NM	Mainly nurses, psychiatric residents, social workers
10	General public hospital	4 PER in large cities in Belgium	NM	NM	NM	NM	NM
11	NM	City with 100,000 inhabitants Catchment: university and industrial town	24 h a day service	NM	NM	NM	NM
12	NM	A: Catchment: part of central city Copenhagen, 2.5 km from PER, population density B: Catchment area: university and industrial town of Oulu, 30 km from PER, population density	Open referral system, 24 h day	NM	NM	A, B: socialized health care system	NM
13	NM	7 PER in San Francisco Bay area	NM	NM	NM	NM	NM
14	University hospital	City with 950,000 inhabitants, 82% African American	Does not provide crisis residential services	NM	Number of hospital admissions per year	NM	Psychiatrists, psychiatric residents, nurses, social workers, mental health technicians
15	See 13						
16	NM	Located in disadvantaged areas in 10 largest cities in US; 82% African American	24 h a day	Part of hospital but not connected to MER	Number of visits per year	NM	Psychiatrists, psychiatric residents, nurses, social workers, mental health technicians
17	University hospital	City of Leuven with 100,000 inhabitants; catchment area of hospital 250,000 people; only hospital with PER	Comprehensive assessment and treatment and disposition plan for each patient; follow-up service	PER program started 1999, automatic enrollment; provides a full range of emergency evaluation, intervention, referral and disposition services for adults in crisis; programs philosophy	NM	Public health care; universal health insurance covering mental health and substance abuse treatment	PER team: 1 supervisor, 2 residents, 1 psychologist, 4 licensed nurses
18	NM	Seattle	24 h a day	NM	Number of visits per year	NM	Psychiatrists, psychiatric residents, nurse practitioners, nurses, social workers
19	General public hospital	City Brussels with 950.000 inhabitants	Special treatment units in total 120 beds, 6 for short-term stays	unrealistic to expect PER to use DSM-IV thoroughly	Number of visits to hospital and PER per year		NM
20	University hospital	A, B: Montreal C, D: Canadian city	C: no observation area	Number of persons served doubled within 2 years		NM	Specialized nurses and psychiatric staff on weekdays; evenings and weekends covered by regular staff, psychiatrists, or psychiatric residents (on call)
21	University hospital	Montreal	NM	NM	Number of visits per year	NM	NM
22	University hospital	Metropolitan region, state and surrounding community	NM	Evaluation by psychiatric residents, no overnight coverage, all evaluations by PR were discussed with child psychiatry attending physician during and before final disposition	Number of visits per year	NM	Psychiatric residents on call daily 8 am – 11 pm,
23	University hospitals	4 PER A: Montreal, catchment area doubled to 180,000 B: Montreal C and D: Kingston (city and metro), no catchment	A: self-contained, secure unit, short-stay observation beds B: no prior medical triage, walk-in clinic, self-contained, secure unit, short-term observation beds, catchment stable C and D: city population increased by 4%; metro population by 10%	NM	NM	NM	NM
24	University hospital	Marseille, 1,000,000 people; description of region	NM	NM	NM	NM	NM
25	University hospital	Detroit	Open access; no formal referral needed; 10 beds short-term stays	NM	Number of visits per year	NM	Study population was staff, but no description beyond study population
26	University hospital	Aarhus, Caucasian, low-wage, highly educated inhabitants	Open access 24 h a day, beds available for short-term stays	NM	Number of visits and hospital admissions per year; self-referral	Public financed by taxes; visit without any charge	Staff is specialized, only works at PER, staff at PER has increased; shifts
27	University hospital	Geneva; catchment of 700,000 located on border	24 h a day	Evaluations and interventions, hospitalization or referral to outpatient service, private psychiatrists, or GP. Initial screening by primary nurse via clinical interview to determine if further in-depth medical or psychiatric. Assessment is needed; description of evaluation	NM	NM	Multidisciplinary team of psychiatrists, nurses, administration staff
28	NM	Busy urban Boston	Different sections	NM	NM	NM	NM
29	University medical center	Large urban New York	NM	NM	Number of visits per year	NM	NM

*NM* not mentioned

### Strengths

The studies’ strengths lay in their transparency concerning the location of the PER, its ownership, and the PER’s capacity. In the studies reviewed, the PER was often located at a university hospital or a public hospital; in one case the PER was community-based [40]. Some studies did not mention the ownership of the hospital, but almost all stated the name of the hospital or city. Most of the articles provided some sort of a description of the PER, such as having open access, 24 h 7 days a week, being available for everyone, or being combined with inpatient or outpatient services. In this way, some sort of context description was provided, which is important in order to correctly interpret and understand the results of the study.

Another strength is that several studies mentioned the total number of visits per year. Particular when provided in combination with number of inhabitants of the city or the catchment area of a PER, it gives a good insight about the size of the hospital and its capacity. In many studies, the number of visits per year was described in the results section, which could be one reason why it is not included in Table 3. One could also draw conclusions about the patients visiting a PER by knowing about the PER’s location, e.g., rural, suburban, or whether the PER is located in an economically disadvantaged area characterized by high unemployment rates or is in an area dominated by a certain ethnographic subgroup, which were mentioned a number of times.

### Weaknesses

The primary weakness of the studies lays in the limited descriptions of organizational structure and processes within PER as well as incomplete descriptions of the local and national contexts in which PER were embedded. Only a limited number of studies mentioned the health care system and its specifics. None of the studies discussed organizational structures such as power and hierarchy or explored the relationship between those structures and the use of resources, be they tangible or intangible. Further, all but one study [33] focused on adult patients and thus excluded adolescents as a group. This is especially a weakness, given that a number of psychotic disorders can be detected at the early age [e.g. 53]. By diagnosing e.g. depression in adolescents, interventions could be implemented earlier and with a preventive purpose [54], which could be beneficial for the patient and increase the efficiency of the service provision.

A further weak point of the studies is that they seldom described the catchment areas, making it impossible to know the total number of visits in relation to population served. Few studies [e.g. 15, 46, 48] have addressed the dynamics within the population in terms of its growth, composition, density, or involvement with other special treatment units. Further, the studies have not addressed the infrastructural aspects such as transportation and accessibility of PER. Finally, the studies have limited discussions on the types of services PER offer, which makes it hard to compare PER across different studies.

### Opportunities

One aspect that could enrich studies of PER would be exploring the team aspect of staff work. Studies in other health-related contexts with a focus on acute and intensive care settings have indicated that teamwork and the dynamics in health care teams are important, given that teams handle complex work assignments better than individuals [55–57]. The complexity of PER and its assignment might thus serve as a golden opportunity in exploring the potential importance of team work and its outcomes. These types of study would be of particular interest in contexts like Swedish PER units, where the staff works in teams with the help of the triage method. Another opportunity for learning more about PER would be to approach them from an organizational perspective, exploring their organizational structure, economic resources, hierarchical relationships between different categories of staff, and how the division of labor between these groups is organized. Exploring these aspects might allow for a more nuanced view of PER and their structuresas well as a better understanding of how those are related to PER’s organizational efficiency and performance. This could be of particular relevance since several studies mention how scarce resources at PER are [e.g. 5, 15]. Exploring staff members’ experiences of their working settings, lived world, and well-being could provide yet another opportunity to better understand how to motivate PER staff and consequently to improve the quality of their work and their level of work satisfaction. Finally, understanding the role of geography (i.e., location in urban or rural areas and communicational and transportation conditions) could shed light on PER’s functioning and the differences that appear between the studies.

### Threats

Again, a threat can be seen in the dominance of the US-based studies, which results in a limited view on the context of PER and their patients. Further, the studies lack information on the methods used within psychiatric emergency care, e.g., in developing countries. Studies tend to focus on a medical/clinical perspective, which leads to a lack of multidisiplinarity. Putting more stress on the nursing/caring perspective within PER could be of importance because the nursing staff is highly involved and are the first to encounter the patient. Another threat is the lack of exploration of geographical aspects of patients vis à vis PER (e.g., rural area vs metropolitan area). The organizational and management structures within PER are also underexplored; knowing more about them could further the understanding of the role and functioning of PER.

## Discussion and conclusion

This literature review posed two research questions: 1) What characterizes frequent visitors at PER in the literature? and 2) What characterizes PER in the literature? Both were explored by the means of a systematic review combined with a SWOT analysis. One aspect that emerged from the literature review is the inconsistent use of the terms “PER” and “frequent visitors.” The broad spectrum of differences in terms can be partly explained by the variation of definitions, variation in different health care settings and welfare systems, geographical and climate differences, and, not least, different populations served. The diversity of conceptualization of PER and frequent visitors represents a challenge. According to McArthur, even a common definition concerning how emergency psychiatric care defines itself needs to be found [8]. In addition, an accepted operational definition of frequent visitors has not yet been proposed [18]. This literature review attempted to provide a first step in developing accepted operational definitions for PER and frequent visitors by surveying the literature on both concepts: PER and frequent visitors. The two are interdependent and thus should both be acknowledged in future definitions of either concept. Though there has been some discussion about what a “true” psychiatric emergency constitute, the basis for all definitions should be found in the urgency of the visitors’ need for care, either as experienced by the person or by others.

In most of the papers, frequent visitors are quantified and objectified, with the studies dealing with frequent visitors at PER based on register data and archival data. There is a lack of qualitative studies investigating the perspective of persons in care in terms of their needs, their satisfaction with PER services, and their life style and living situation. Such studies are needed in order to provide appropriate support and help to frequent visitors and might identify whether the type of help needed could be offered outside of PER. The studies are empirically driven and do not seek to establish models or theories. One way forward would be to look at the findings of the studies and to try to further develop existing models and eventually create new ones. This would also allow for learning more about, e.g., frequent visitors’ attitudes and behaviors by applying existing models. One such model could be, e.g., the Tidal model [19, 58], which was developed for psychiatric care settings. Another possibility for seeking explanation for the results would be the application of existing theories, e.g., Giddens’ structuration theory that poses that society is based on social actions and should be understood in terms of agency (relationship with other people based on interactions) and structure (rules and resources). Agency and structure exist in duality (i.e., they involve reciprocity between actors and collectives) [59]. This review revealed that the studies focus mainly on the characteristics of the agent (the frequent visitors) and do not do enough to take into account the implications of PER and the challenges PER faces (structural aspects) when caring for frequent visitors. In order to understand the agency and the structure, one needs to study their interactive nature. Applied here, it means that future studies should focus on understanding the role of PER for frequent visitors and the role of frequent visitors for PER, which so far has not found its way into the literature [cf. 18].

The opportunity and threat discussions further revealed the need for studies that address person-centeredness. Person-centered frameworks have recognized the important role of the care environment with its hinders and facilitating roles [60], thus context needs to be considered more when investigating frequent visitors at PER. Such investigations should include physical setting, organizational systems, professional competencies, human relationships, and hierarchies [61], as well as the interpersonal context [20]. PER provides a unique context for caring processes to occur and for interactions between staff and patient. The first encounters between frequent visitors and staff and how staff interacts with and cares for them is part of the therapeutic relationship [e.g. 62, 63–65]. Concepts of transference and countertransference might need to be taken into account, in particular in this setting.

Further the literature has shown that frequent visitors psychological and service needs are complex, vary from patient to patient and between the contexts. Thus, understanding patients’ needs better would allow for better fitted and tailored interventions that also strive for continuity. The latter could only be achieved by collaboration with external actors.

In reviewing the literature, it became apparent that PER continue to face challenges, given their fast-paced environments, and insufficient time for staff to provide giving diagnoses and care while serving acutely ill, vulnerable persons that need interhuman and interpersonal interaction. Instead, the literature is in agreement when addressing PERs’ frequent visitors as ‘hard to treat’ and ‘difficult patients’ [20, 47, 66–68] or those that cannot profit from psychiatric treatment [2]. Little information is revealed about how frequent visitors could profit from the newly gained insights of the studies. Future research would benefit from applying the aforementioned theories or models or other person-centered frameworks that stress acknowledging the patient as an equal partner in the health care process. Such theories or models also conceptualize the context and the person(s) of the studies, which might lead to the development of the former and improved care for the latter.

## Footnotes

1 SWOT is one of the most known approaches used for analysis of a company’s strategic position being the acronym for Strengths, Weaknesses, Opportunities and Threats. The idea of ensuring a fit between the external situation (threats and opportunities) and own internal qualities (strengths and weaknesses) has shown to be very popular ([32].Hill, T. and R. Westbrook, *SWOT analysis: it’s time for a product recall.* Long range planning, 1997. **30**(1): p. 46–52.) not only in the field of business administration but also is widely being used for any kind of strategic planning or examination of projects, organizations, companies or other ventures.
